# Implementation Determinants of Integrated Tuberculosis and Diabetes Care in South Asian Association for Regional Cooperation (SAARC) Countries: A Systematic Review

**DOI:** 10.5334/ijic.9882

**Published:** 2026-06-24

**Authors:** Saima Aleem, Saima Afaq, Suneel Gill, Bilal Ahmad, Zunaira Michael, Rida Zarkaish, Zohaib Khan

**Affiliations:** 1 Institute of Public Health & Social Sciences, Khyber Medical University, Peshawar, Pakistan; 2 Office of Research Innovation and Commercialization, Khyber Medical University, Peshawar, Pakistan

**Keywords:** tuberculosis, diabetes mellitus, comorbidity, integrated care, implementation determinants, barriers and facilitators, health systems

## Abstract

**Background:**

Tuberculosis (TB) and diabetes mellitus (DM) represent a growing syndemic in low- and middle-income countries (LMICs), particularly across South Asia. The bidirectional relationship between these diseases exacerbates health outcomes and increases system burdens. Although the World Health Organization has advocated for integrated management of TB and DM, implementation remains inconsistent across the SAARC region. This systematic review aims to identify and analyse implementation determinants of integrated TB and DM care in SAARC countries.

**Methods:**

We conducted a systematic review following PRISMA 2020 guidelines. searching MEDLINE (via Ovid), EMBASE, Web of Science, Cochrane CENTRAL, and CINAHL for peer-reviewed studies. Grey literature was sourced from Google Scholar and citation search. Four reviewers independently screened title, abstract and full text using Rayyan. Using a structured Excel form, two reviewers extracted data. Quality assessment was conducted by using Mixed Methods Appraisal Tool (MMAT). A narrative synthesis was conducted in line with SWiM guidelines to categorize implementation determinants as barriers or facilitators.

**Results:**

Ten studies met the inclusion criteria and were conducted across five SAARC countries: India (n = 7), Pakistan (n = 1), Bangladesh (n = 1), and Sri Lanka (n = 1). Identified facilitators included political commitment, use of digital tools, and training of healthcare workers. Barriers encompassed inadequate infrastructure and finances, workforce shortages, lack of standardized guidelines and fragmented vertical health systems.

**Conclusion:**

Integrated TB-DM care in the SAARC region remains at an early developmental stage, with most efforts limited to pilot projects or small-scale screenings. Despite political and institutional recognition of the dual burden, scale-up is constrained by systemic barriers, resource gaps, and lack of evidence-informed implementation strategies. Future efforts should prioritize system-wide integration guided by implementation frameworks, standardized protocols, and investment in workforce and infrastructure to achieve sustainable impact.

**Systematic review registration:**

PROSPERO registration number: CRD42025644263.

## Introduction

Tuberculosis (TB) and diabetes mellitus (DM) represent growing dual epidemics with significant public health implications, particularly in South Asian countries [1] where both conditions are highly prevalent with three countries accounted for 35.8% for global total for tuberculosis include India (26%), Pakistan (6.3%), and Bangladesh (3.5%) [2] and diabetes prevalence in this region increase to 22.30% in 2020-2024 [3]. TB infection can impair glycaemic control in individuals with diabetes and potentially increase the risk of diabetes up to 30% [4], while diabetes is also an independent risk factor for tuberculosis [5] and diabetic patients have two to four-fold higher risk of developing TB [6]. This complex bidirectional relationship necessitates integrated care and management approaches where integration is specified as a type of managerial or operational transformation to health systems to combine interactions, production, management, and organisation of TB and diabetes services activities, and various forms of diabetes and TB services, operation programmes, or operations brought together to guarantee and possibly maximise overall outcomes in patients with diabetes and with TB [7].

The World Health Organization (WHO) and the International Union Against Tuberculosis and Lung Disease (The Union) introduced the “Collaborative Framework for Care and Control of Tuberculosis and Diabetes” in 2011, promoting bidirectional screening and integrated management strategies [8]. Given the intersecting epidemiology of TB and DM, integrated care that addresses both conditions concurrently have emerged as a promising approach to improve clinical outcomes, reduce healthcare costs, and streamline service delivery in many countries.

Tanzania’s Adaptive Disease Control Expert Programme (ADEPT) used TB-DM as case study for integrated care [9], South Africa’s USAID TB CARE II supported guideline-based training and diabetes screening equipment for TB services [10], and the government of Malawi, adopted an integrated approach for non-communicable diseases into infectious diseases [11,12]. While the WHO and The Union framework has guided the development of national policies, the implementation of integrated care for TB-DM comorbidity is complex, involving multiple levels of the health system, from policy and governance to health facility, clinical and community-based services [13,14,15] and in several high-burden countries including India, Pakistan and Bangladesh, the implementation of integrated TB-DM care faces numerous challenges that influence its effectiveness in real-world settings [14,16].

South Asia, comprising Afghanistan, Bangladesh, Bhutan, India, Maldives, Nepal, Pakistan, and Sri Lanka, is densely populated sharing similar geopolitical environments, socioeconomic conditions, burden and pattern of diseases and health infrastructure. As a result of similar conditions, South Asian Association for Regional Cooperation (SAARC) was founded in 1985 to help these eight countries collaborate on social, economic, and health issues. These SAARC member states continue to experience a high burden of TB alongside a rapid rise in diabetes prevalence driven by urbanization, lifestyle shifts, and demographic changes. The co-occurrence of TB and DM constitutes a syndemic, with each condition aggravating the other and complicating diagnosis, treatment, and outcomes. To inform context-specific policy and programme development, it is essential to understand the implementation determinants both barriers and facilitators that influence the integration of TB and DM care within these settings. These determinants may be internal (e.g., provider readiness, infrastructure, training) or external (e.g., political commitment, funding, community engagement), and their identification is critical for designing effective, scalable, and sustainable interventions.

Given the co-occurrence of high TB burden and rising diabetes prevalence in SAARC countries [17,18], together with their bidirectional interaction that increases comorbidity and complicates care pathways [19,20], it is critically important to review the published literature from SAARC countries to understand how integrated TB-DM care is being approached in diverse but comparable settings. Synthesizing region-specific evidence allows for the identification of contextually relevant barriers and enablers, enabling mutual learning and informing regional strategies. Moreover, the SAARC Tuberculosis and HIV/AIDS Centre (STAC), a regional body established to coordinate TB control efforts, offers a potential platform to translate such evidence into collaborative regional action [21].

Despite growing interest in the TB-DM interface, there remains a lack of synthesized evidence on how integrated care is being implemented across the SAARC region. This systematic review addressed this knowledge gap by identifying and analysing the determinants that shape the implementation of integrated DM services in existing TB programs. By doing so, it aimed to ultimately inform policymakers, program managers, and practitioners working to improve integrated service delivery and accelerate progress toward TB and non-communicable disease (NCD) control goals. Following the Synthesis Without Meta-analysis (SWiM) guidelines [22], we present a comprehensive analysis of the implementation determinants, barriers, facilitators, documented in the current literature reflexively.

## Methods

### Design and Reporting

We had developed this systematic review protocol using Preferred Reporting Items for Systematic Reviews and Meta-Analyses Protocols (PRISMA-P) 2015 checklist and conducted this systematic review following the Preferred Reporting Items for Systematic Reviews and Meta-Analyses (PRISMA) guidelines. The protocol was registered with PROSPERO (registration number CRD42025644263) [23].

### Outcome Measures

For this review, the primary outcomes of interest were implementation determinants of integrated TB-DM care in SAARC countries. An implementation determinant is a factor that influences the success, process, or outcomes of implementation in a particular context. For this review, implementation determinants either enabling or hindering the implementation of integrated care were identified by employing determinant framework [24]. The Consolidated Framework for Implementation Research (CFIR) provides a comprehensive theory-informed structure for systematically identifying, organizing, and interpreting the complex determinants that influence implementation processes across diverse healthcare settings [25]. These determinants can be internal (e.g., organizational readiness, staff training) or external (e.g., policy environment, community support).

### Data Searches

Data was searched from the EMBASE and MEDLINE via Ovid, Cochrane CENTRAL, CINAHL and Web of Science from 2011 to 2025. We limited the search to studies published from 2011 onwards because the WHO and The Union Collaborative Framework for Care and Control of Tuberculosis and Diabetes was released in 2011, establishing the key policy basis for bidirectional screening and integrated TB–DM service delivery. Non-English papers were excluded for which translation could not be obtained during the timeframe of the review. Search was conducted using the search string mentioned in Annexure A.

### Study Selection

All primary research published in the English language, comprising quantitative studies, qualitative studies, and mixed-methods studies were included in the systematic review to comprehensively identify the implementation determinants of integrated Tuberculosis (TB) and Diabetes Mellitus (DM) care. Studies were included if they focused on integrated care models, services, or interventions that addressed both TB and DM concurrently. The determinants could have been stated explicitly or inferred through descriptions of linked, coordinated, or combined service delivery for both conditions. Studies that focused exclusively on either TB or DM were excluded unless they clearly discussed integration with the other condition.

To be eligible, studies were required to report on factors influencing the implementation of integrated TB-DM care. These included barriers, facilitators, enablers, challenges, or any contextual or system-level determinants affecting implementation. Both explicitly stated implementation determinants and those inferred from analysis or discussion were considered.

### Data Extraction and Quality Assessment

Two reviewers (SAL & SG) independently extracted data using a structured form developed in Microsoft Excel. We captured bibliographic details, study characteristics (design, setting, population), implementation framework(s) used, and detailed information on implementation determinants (barriers, facilitators, and proposed remedial strategies).

Quality assessment was conducted using the Mixed Methods Appraisal Tool (MMAT) for mixed-methods studies, qualitative studies, and quantitative studies. Two reviewers independently assessed quality, with disagreements resolved through discussion.

The quality for this systematic review was also ensured following the standard reporting guidelines including PRISMA for abstract [26] and PRISMA checklist [26,27] attached in supplementary file 2 (Annexure D & E).

### Data Synthesis

We used a narrative synthesis approach following the Synthesis Without Meta-analysis (SWiM) guidelines [22]. Given the heterogeneity of study designs, contexts, and reported outcomes, meta-analysis was not appropriate. Instead, we conducted a theoretically-informed synthesis reflexively using the Consolidated Framework for Implementation Research (CFIR) [28].

**Figure 1:**
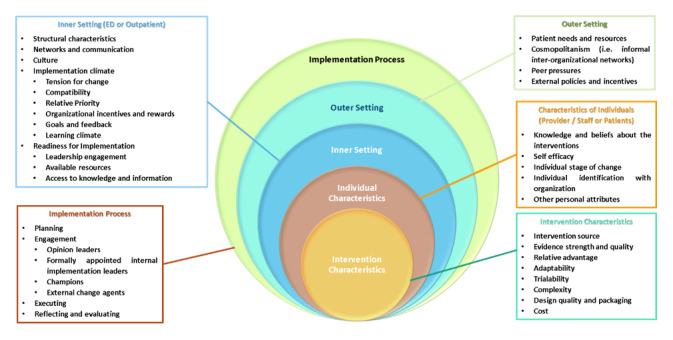
Domains and Constructs of Consolidated Framework for Implementation Research [20].

The CFIR provides a comprehensive structure for understanding implementation determinants across five domains as presented in Figure 1:

1. **Innovation Domain:** Characteristics of integrated care intervention.
2. **Outer Setting Domain:** External influences on implementation.
3. **Inner Setting Domain:** The internal organizational context.
4. **Individual Domain:** Characteristics of the people involved in implementation.
5. **Implementation Process Domain:** Activities undertaken to facilitate implementation.

By mapping implementation determinants to these domains, we provided a comprehensive understanding of the factors influencing TB-DM integrated care across different contextual settings.

## Results

### Literature Search

**Figure 2:**
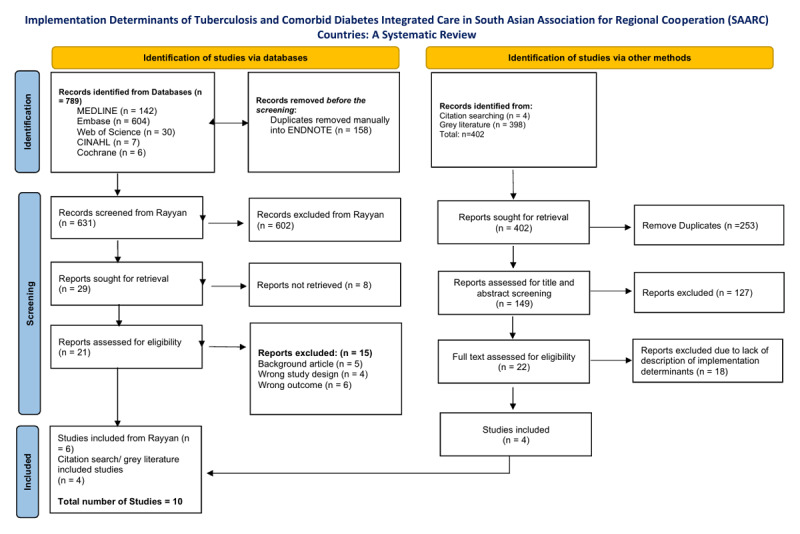
PRISMA flow diagram for selection of articles for systematic review of implementation determinants of integrated TB diabetes care in SAARC countries.

We identified 789 records from data bases and additional 402 from other sources as mentioned in PRISMA Flow Diagram (Figure 2). After initial title and abstract and full text screening, we included 10 studies comprising quantitative (n = 5), qualitative (n = 1) and mixed method (n = 4).

### Characteristics of Selected Studies

A total of 10 studies conducted in SAARC countries were included in this systematic review and their characteristics are shown in Table 1. The included studies were conducted across five SAARC countries: India (n = 7), Pakistan (n = 1), Bangladesh (n = 1), Sri Lanka (n = 1). No study as per the research question was retrieved from Bhutan, Maldives and Afghanistan.

**Table 1:** Study characteristics of included studies.


AUTHORS AND YEAR	TITLE	COUNTRY	JOURNAL	STUDY SETTING	SAMPLE SIZE	STUDY POPULATION CHARACTERISTICS/INCLUSION CRITERIA	AIMS & OBJECTIVES	STUDY DESIGN

Basir et al; 2019 [29]	Operationalization of bi-directional screening for TB and diabetes in private sector healthcare clinics in Karachi, Pakistan	Pakistan	BMC Health Services Research	Private clinics	450,385 individuals tested (18,109 with presumptive DM and 90,137 with probable TB).	Clinic attendees screened for TB and/or DM; bacteriologically confirmed TB referred for DM testing; pre-DM/DM referred for CXR and Xpert	Feasibility of bi-directional TB–DM screening in private sector using a social business approach	Cross-sectional

Swain et al; 2021[4]	Factors Affecting Diabetes Management among TB-Diabetes Comorbid Patients in Udupi District	India	Indian Journal of Community Medicine	Public healthcare facilities under the Revised National TB Control Program (RNTCP)	Quantitative Study: 154 patients Qualitative Study: 10 Medical Officers	All TB-DM comorbid patients registered in NIKSHAY (2018–2019); purposive sample of MOs for IDIs	Identify factors and barriers influencing diabetes management among TB-DM patients	Mixed-method cross-sectional with convergent parallel design.

Joshi et al; 2022 [30]	Integrated Management of Diabetes and TB in Rural India – Results from a Pilot Study	India	Frontiers in Public Health,	10 Primary Health Centers (PHCs) with co-located DOTS centers.	120 patients (57 intervention, 63 control)	Adults (≥18) with recent TB diagnosis (≤4 months)	Field-test a multidisciplinary, digitally supported TB–DM integration model (SMARThealth); assess feasibility and effect	Cluster RCT with mixed-methods evaluation

Satyanarayana 2013 [8]	Screening of patients with TB for diabetes mellitus in India	India	Tropical Medicine & International Health	8 tertiary hospitals; >60 PHIs across 8 TB units	8269 TB patients screened for diabetes	Newly registered/confirmed TB patients	Assess feasibility and outcomes of DM screening within routine TB care	Prospective observational implementation study

Koya et al; 2022 [31]	TB and Diabetes in India: Stakeholder Perspectives on Health System Challenges and Opportunities for Integrated Care	India	Journal of Epidemiology and Global Health	Program staff and providers across two states	33 participants (program officials, partners, MO, private clinician; two FGDs with HCWs))	Stakeholders from TB–DM programs (public/private)	Explore system-level challenges and opportunities for TB–DM integration	Qualitative

Anand et al; 2018 [32]	Integrating screening for non-communicable diseases and their risk factors in routine TB care in Delhi, India: A mixed-methods study	India	PLoS One	DOTS centers at two medical institutions, Delhi.	410 TB patients and HCP interviews	TB patients ≥ 20 years on treatment; participating HCPs	Evaluate feasibility/acceptability of 2-stage integrated NCD (incl. DM) screening in TB care	Mixed-methods

Majumdar et al; 2019 [33]	TB-diabetes screening: how well are we doing? A mixed-methods study from North India	India	Public Health Action	Public healthcare facilities, Sonipat, India	652 TB patients	All TB cases (Nov 2016–Apr 2017) and DM patients at NCD clinics	Estimate TB diabetes patients’ proportions screened both ways; identify facilitators/barriers and solutions	Mixed methods

Rafi et al; 2024 [34]	An approach to integrated management of diabetes in TB patients: Availability and readiness of the health facilities of Bangladesh	Bangladesh	PLoS One	National facility survey,	1596 health facilities	Facilities providing TB diagnosis/treatment (OPD and inpatient)	Assess availability and readiness of TB care centres for DM management	cross-sectional survey (health-facility readiness)

Rajapakshe et al; 2015 [35]	Screening patients with TB for diabetes mellitus in Ampara, Sri Lanka	Sri-Lanka	Public Health Action	chest clinic in the Ampara, Sri Lanka	112 TB patients	Registered TB patients; DM defined by FBG thresholds; new DM/IFG enrolled to care	Determine prevalence and feasibility of DM screening among TB patients	cross-sectional pilot study

Kunjathur et al; 2025 [36]	Diabetes among tuberculosis patients in Bengaluru is alarming: Time to tackle it efficiently	India	Indian Journal of Tuberculosis	32 TB units; 147 DMCs; Bengaluru	17,052 presumptive TB cases; staff interviews across 8 TB units	Presumptive TB cases screened; qualitative interviews with NTEP staff	Examine TB–DM association; feasibility and operational barriers/facilitators for screening	Cross-sectional with qualitative component


### Quality Assessment

Systematic reviews which combine, quantitative, qualitative an mixed method studies are useful in addressing complex evaluation research questions [37,38,39] We conducted quality assessment in this systematic review by using the Mixed Methods Appraisal Tool (MMAT) considering the diverse range of methodological designs of the included studies [40]. The MMAT is specifically designed to appraise the methodological quality of multiple types of empirical research using a single, coherent framework. Unlike other tools that are restricted to a specific study type, MMAT offers distinct sections tailored for qualitative research, randomized controlled trials, non-randomized studies, quantitative descriptive studies, and mixed-methods studies. Additionally, MMAT’s structured criteria enable the identification of methodological strengths and limitations, thereby improving the transparency and rigor of quality appraisal.

In the present systematic review, quality assessment was conducted by two reviewers (SAL, SG). The included studies in this systematic review were categorized into ‘qualitative’ (n = 1) (Koya et al., 2022), ‘quantitative descriptive’ (n = 5) (Basir et al., 2019; Rafi et al., 2024; Satyanarayana et al. (2013); Rajapakshe et al., 2015; Kunjathur et al., 2025), and ‘mixed methods’ (n = 4) (Swain et al., 2021; Joshi et al., 2022; Anand et al., 2018; Majumdar et al., 2019). Overall, study quality was moderate to high: the qualitative study met all MMAT criteria (5/5); most quantitative descriptive studies satisfied the majority of MMAT criteria (typically 4/5), with one lower-scoring study due to potential non-response/attrition concerns; and all mixed-methods studies demonstrated an appropriate rationale and integration of qualitative and quantitative components, with component strands meeting MMAT criteria. Individual assessment of each study is added in Supplementary file 1 Annexure C.

### Implementation Frameworks for TB-DM Integration

#### Global and National Policy Frameworks

The WHO–Union Collaborative Framework is the primary guide for TB–DM integration, emphasising bidirectional screening, joint coordination, and health system monitoring [8].

In India, integrated management is guided by National Framework in Joint TB-Diabetes Collaborative Activities (2017) under the Revised National Tuberculosis Control Programme (RNTCP) and National Programme for Prevention and Control of Cancer, Diabetes, Cardiovascular Diseases, and Stroke (NPCDCS) [4]. However, evidence suggests that policy guidance does not automatically translate into routine practice; Swain et al. (2021) reported persistent operational challenges at facility level despite the framework [4].

#### Bidirectional Screening Approaches

Bidirectional screening is central to TB–DM integration, requiring systematic screening for TB among people with diabetes and for diabetes among TB patients. In Karachi, Pakistan, this was operationalised in private clinics through community health workers using an algorithm aligned with national guidance and International Diabetes Federation (IDF) recommendations [29]. In India, embedding random and fasting glucose testing at TB diagnosis detected previously undiagnosed DM and demonstrated feasibility at scale [8].

#### Community-Based Integrated Care Models

Joshi et al. (2022) piloted an innovative rural community-based TB-DM integration approach at primary care, utilizing digital health solutions [30]. Their model adapted the SMARThealth application to include TB, DM, and cardiovascular disease (CVD) screening and management protocols, enabling task sharing between Auxiliary Nurse Midwives (ANMs) and primary health centre (PHC) physicians.

#### Facility-Based Integration Models

Facility-based integration models co-locate and coordinate TB-DM services within existing health facilities. In North India, Majumdar et al. (2019) highlighted the challenges and enablers of bidirectional screening in facilities, including workforce constraints, knowledge gaps, and coordination issues [33].

#### Implementation Determinants through the CFIR Lens

For this systematic review, we used CFIR to embed the implementation determinants including both barriers and facilitators as presented in Tables 2, 3 and 4 as CFIR offered a theory-informed framework to effectively identify, organize and interpret determinants specific to SAARC countries which have similar health system structures, operational constraints, and vertical programmatic approaches to address TB-DM integration.

**Table 2:** Inner Setting Barriers to TB-DM Integrated Care.


BARRIER	WHAT IT LOOKS LIKE IN SAARC SETTINGS	WHY IT MATTERS (MECHANISM)	KEY STUDIES

Structural characteristics	Rural infrastructure gaps; few trained NCD staff; high ANM caseloads; fragmented TB–NCD pathways	Low feasibility; weak continuity; need for task-sharing and clearer pathways	(30)

Workforce & workload	Understaffing; limited training; added screening duties overload DOTS; limited male cadres	Slower screening: counselling/follow-up crowded out; reduced engagement with male patients	(33, 32, 30, 4)

Networks & communications	Weak RNTCP–NPCDCS coordination; unclear referral channels	Missed transitions to DM care after TB treatment ends; fragmentation	(4)

Readiness for implementation	Lack of staff and guidelines, diagnostics and equipment gaps; scarce supplies at the primary level	Constraints sustained bidirectional screening and DM management	(34, 33)

Resource limitations	Unavailable tests and diagnostics; equipment shortages; supply gaps; out-of-pocket expenses for patients	Delays and drop-off, cost, and distance suppress diagnostic completion	(36, 8, 32, 29)

Reporting & monitoring	Paper records; non-standard Diabetes reporting in TB services; unclear post-TB diabetes protocols	Inconsistent documentation, tracking, and follow-up	(8, 32, 4)


**Table 3:** TB-DM integrated care barriers mapped to CFIR domains.


AUTHORS AND YEAR	BARRIERS CHARACTERISTICS	CFIR CONSTRUCT				

		INNOVATION	OUTER SETTING	INNER SETTING	INDIVIDUAL	IMPLEMENTATION PROCESS

**Basir et al; 2019 [29]**	Operational constraints	Systemic and financial constraints	Cost barriers	Operational constraints	Asymptomatic patients	Detection gap

**Swain et al; 2021 [4]**	Patient and health care provider related barriers	Lack of follow up protocol for diabetes after end of TB treatment	Limited support	Understaffed facilities	Alcoholic patients	Protocol confusion

**Joshi et al; 2022 [30]**	Workload	Not mentioned	Poor coordination	High workload	Limited training	Not mentioned

**Satyanarayana 2013 [8]**	Workload pressure	Difficulty in collation of a total denominator for the study	Weak referral	Equipment shortage	Limited awareness	Workload pressure

**Koya et al; 2022 [31]**	Perception barriers	Not mentioned	Public-private gap	Ownership issues	Training needs	Case detection barriers

**Anand et al; 2018 [32]**	Workload concerns	Not mentioned	Not mentioned	Inadequate supplies	Inadequate skills	Not mentioned

**Majumdar et al; 2019 [33]**	Patient awareness	Non-availability	Staff shortage	Resource constraints	Awareness deficit	Knowledge gaps

**Rafi et al; 2024 [34]**	Knowledge gaps	Diagnostic tools	Policy gaps	Fragmented structure	Staff knowledge gaps	Poor coordination

**Rajapakshe et al; 2015 [35]**	Inadequate addressed TB co-morbidities esp diabetes	Limited diagnostics	Cost effectiveness	Follow-up issues	Stress inducted hyperglycaemia	Operational challenges

**Kunjathur et al; 2025 [36]**	Operational challenges	Appropriate diagnostic facilities	Lack of x rays leading to referrals	Lack of availability of tests that were needed to be done for the patients	Patients not complaint to FBS appointments and timings Lack of awareness among patients for screening	Operational challenges


**Table 4:** Implementation Facilitators for TB-DM Integrated Care.Implementation Facilitators for TB-DM Integrated Care.


AUTHORS AND YEAR	FACILITATORS CHARACTERISTICS	CFIR CONSTRUCT				

		INNOVATION	OUTER SETTING	INNER SETTING	INDIVIDUAL	IMPLEMENTATION PROCESS

**Basir et al; 2019 [29]**	Private sector involvement	Preventative intervention	Cost-free services	Operationalizing CMW role with private service providers	Encourage screening	Selection bias

**Swain et al; 2021 [4]**	Single-point care	Re-screening effectiveness	Not mentioned	Care continuity	Not mentioned	Structured counselling

**Joshi et al; 2022 [30]**	Existing trust	Digital support	Policy alignment	Embedded care	Trust relationship	Supportive supervision

**Satyanarayana 2013 [8]**	Policy support	Minimize inconvenience	Strong policy	Existing infrastructure	Positive response	Not mentioned

**Koya et al; 2022 [31]**	Healthcare infrastructure	Existing frameworks	Effective communication	Strong infrastructure	Not mentioned	Not mentioned

**Anand et al; 2018 [32]**	Simple tools	Simple tools	Not mentioned	Short wait times	Simple tools	Short wait times

**Majumdar et al; 2019 [33]**	Positive attitude	Decentralized testing	Positive attitude	Decentralized facilities	Positive attitude	Training providers

**Rafi et al; 2024 [34]**	Policy support	Existing clinics	Policy support	Resource leverage	Community workers	Extended roles

**Rajapakshe et al; 2015 [35]**	Implementation feasibility	Global alignment	National support	Integration success	High enrolment	Feasibility demonstrated

**Kunjathur et al; 2025 [36]**	Existing strong human resource and teams	Screening was easy to conduct	Policy support	Availability of medicines	Strong team	Feasibility demonstrated for screening


### Innovation Domain

#### Facilitators

##### Innovation Source, Evidence Strength, Relative Advantage and Adaptability

Across studies, the innovation was easier to adopt when it was anchored in recognised guidance, perceived to add value over fragmented care, and fitted into routine workflows without major disruption. Alignment with India’s National Framework for Joint TB–Diabetes Activities (2017) and the WHO Collaborative Framework strengthened legitimacy and acceptability [4,30]. Evidence of feasibility and yield further supported uptake; for example, embedding diabetes screening within routine TB care demonstrated operational viability [8].

Integrated models outperformed fragmented care by enabling earlier DM detection in TB patients, improving glycaemic control, continuity, and follow-up [4]. Adaptability helped when screening was delivered through simple, workflow-compatible steps, when routine glucose testing was embedded within TB services, and when digital decision support enabled task-shifting at primary care level [8,30,32,35].

#### Barriers

##### Innovation Cost

A recurring barrier was sustainability, as initiatives often relied on project or research funding rather than routine programmatic resources, limiting scale-up when external funding ends [8,29].

### Outer Setting Domain

#### Facilitators

##### External Policies, Incentives, Partnerships & Connections

Policy alignment and enabling governance were consistent facilitators of TB–DM integration, particularly where national guidance was supported by local leadership and accountability structures [4]. Evidence also indicates that political commitment at sub-national level can shape implementation intensity and consistency; for example, stronger state leadership enabled more effective integration compared with settings where political support was weaker [31]. Similarly, cross-sector partnerships expanded reach and uptake: in Karachi, private clinics working with community health workers strengthened linkages to diagnostic services and improved the operationalisation of bidirectional screening in routine practice [29].

#### Barriers

##### Fragmented Health Systems, Patient Needs and Resources

Fragmented TB–DM service structures limited continuity of integrated care, particularly after TB treatment completion, due to weak referral pathways and poor coordination between TB and NCD programmes. Swain et al. (2021) reported low referral from TB services to diabetes care after treatment completion [4], while Joshi et al. (2022) similarly noted disruption of follow-up where TB–NCD coordination was weak [30]. Koya et al. (2022) highlighted decentralisation and stronger district coordination to operationalise bidirectional screening and management [31]. The fragmented system gaps compound with patient-related social and behavioural factors, including alcoholism, migrant status, old age, and lack of family support [4], and low awareness of bidirectional screening further limits engagement, with patients deferring to clinicians rather than requesting tests [33].

### Inner Setting Domain

#### Facilitators

##### Relational Connections & Coordination

Relational coordination within facilities supported integration when TB and DM services were organised to reduce fragmentation and strengthen continuity. Co-locating TB and DM management in the same facility enabled single-point care, improving follow-up and adherence to treatment plans [4]. Integration was further strengthened when workflows were aligned with routine TB service delivery, for example, aligning diabetes follow-up with DOTS visits reduced duplication and streamlined care within resource constraints [30]. Similarly, embedding screening into existing TB care workflows minimised disruption and improved provider acceptability [32].

##### Service Readiness, Communication, and Workforce Capability

Across the included studies, integrated TB diabetes care was easier to deliver when facilities were ready to implement it, and staff were supported to do it well. Facility readiness, including adequate staff, equipment and diagnostics, medicines, and clear guidelines, shaped service delivery capacity, and readiness assessments helped identify and address gaps in infrastructure for integrated care [34]. Similarly, strong communication networks between facilities, TB and diabetes programs supported by outreach and counselling, also helped sustain screening and follow-up and close operational gaps [36].

Training and capacity building further strengthened implementation. Joshi et al. (2022) delivered a two-day ANM training on use of the SMARThealth app, counselling, and lifestyle advice, reinforced through supervision and mentoring [30]. Anand et al. (2018) and Majumdar et al. (2019) recommended ongoing training and professional development for healthcare providers as an effective way to enhance implementation of integrated screening [32,33].

##### Barriers

Across the ten studies, inner-setting barriers consistently clustered around workforce and workload constraints, weak TB–diabetes service interfaces, and gaps in facility readiness and resources. These barriers jointly undermined referral continuity, sustained screening, and the consistency of counselling and follow-up (4, 8, 29, 30, 32–34, 36) as presented in Table 2.

#### Individual Domain

##### Facilitators

###### Providers buy-in, Role Clarity, and Patient Acceptability

Across studies, individual-level facilitators centred on provider buy-in and confidence to deliver integrated workflows, supported by team functioning and role clarity. Where teams were cohesive and human resources were sufficient, task-sharing enabled more consistent screening and follow-up [36]. Providers also reported embedding diabetes screening within TB care despite resource constraints, reflecting proactive adoption when staff were supportive [32], and positive staff attitudes similarly enabled bidirectional screening even when knowledge and resources were not optimal [33]. Acceptability was further strengthened by trusted ANM–patient relationships that improved counselling uptake and adherence [30], and by clearly defined roles among TB staff, with brief training helping translate policy into routine practice [4]. On the patient side, willingness to be screened and perceived value of screening supported implementation [8,32], and structured end-of-treatment counselling improved diabetes management behaviours by reinforcing follow-up and lifestyle actions [4].

##### Barriers

###### Knowledge, Beliefs, Providers and Patients Factors

Barriers at the individual level were driven by knowledge and belief gaps and constraints on changing practice, alongside patient-level practical barriers. Several studies reported limited provider knowledge of TB–DM comorbidity and bidirectional screening protocols [32,33], and diabetes clinicians’ reluctance to add TB screening responsibilities because of workload pressures [8]. Similarly, patient-related barriers reduced screening completion, including non-attendance for fasting blood samples and reporting times [36].

Studies highlighted the low patient awareness about screening needs, dependence on doctors’ instructions [33], and inn settings without advanced diagnostics, interpretation challenges such as possible stress-induced hyperglycaemia may complicate follow-up decisions [35]. Across studies, persistent training and capacity gaps were repeatedly highlighted as a core barrier to effective integration [31,32,34].

#### Implementation Process Domain

##### Facilitators

###### Planning (Implementation planning and operationalisation tools)

Across implementations, planning that translated national or global guidance into operational workflows and practical tools supported smoother integration within routine TB services. In India, Joshi et al. (2022) demonstrated that a structured planning process, summarising guidance, developing programmable algorithms, clinical validation, and user-interface development for the SMARThealth application helped operationalise integrated delivery in primary care settings [30]. Similarly, Satyanarayana et al. (2013) showed that adapting WHO–Union collaborative framework protocols to existing TB care pathways enabled structured implementation within routine programme workflows [8].

###### Engaging (Stakeholder engagement and co-design)

Integration was most feasible and acceptable when implementation was co-designed with stakeholders across levels, including frontline providers, programme managers, and policymakers, to align incentives, clarify roles, and formalise referral and monitoring pathways [31]. Evidence also highlights the importance of public–private and community linkages for operationalising engagement in practice. In Karachi, partnering private clinics with community health workers (CHWs) and actively involving patients expanded screening reach and completion while also identifying practical access barriers (e.g., fees, travel) that informed workflow adaptations (e.g., on-site triage and linkage) [29]. Collectively, these findings argue for early, structured engagement across public–private actors, stakeholders and patient groups to improve adoption, fidelity, and context-specific integration.

###### Reflecting and Evaluating (Monitoring, feedback, and learning)

Across implementations, reflection and evaluation ranged from structured process evaluation to descriptive reflection on existing practice but consistently served to identify feasibility constraints and inform improvement opportunities. Joshi et al. (2022) used a formal process evaluation approach, incorporating feedback from ANMs and patients and tracking quantitative indicators (e.g., screening rates, follow-up adherence, and glycaemic control) to assess feasibility, acceptability, and implementation barriers [30]. Swain et al. (2021) systematically documented gaps, barriers, and enablers in routine TB–DM collaborative care to inform strengthening of integration [4]. These findings indicate that incorporating structured monitoring and feedback, whether through formal evaluation or systematic reflection, supports iterative learning and refinement of integrated TB–DM care models.

Tables 3 and 4 show that implementation determinants of integrated TB-DM care were distributed across all five CFIR domains, indicating that integration is shaped by interacting health-system, provider, patient, and implementation-process factors. The most frequently reported barriers were located in the inner setting and individual domains, including workforce shortages, high workload, limited training, weak referral coordination, inadequate diagnostic supplies, and low provider or patient awareness. Outer-setting barriers such as fragmented public-private systems, policy gaps, and financial constraints further limited continuity of care and scale-up. In contrast, key facilitators included policy support, existing TB infrastructure, co-located or single-point care, digital decision-support tools, community health worker involvement, positive provider attitudes, structured counselling, and supportive supervision. Overall, the evidence suggests that sustainable TB-DM integration in SAARC countries requires strengthening facility readiness, workforce capacity, referral mechanisms, and implementation monitoring while building on existing policy frameworks and service platforms.

## Discussion

This narrative synthesis revealed several key insights regarding the implementation determinants of integrated DM care into existing TB programs in SAARC countries as TB platforms provide a pragmatic entry point for strengthening person-centred chronic care in high-burden settings. Although global and national policy frameworks provide essential guidance, significant gaps exist between policy and implementation. The WHO-Union Collaborative Framework and various national adaptations offer valuable structures; however, operational guidelines often fail to address the complexities of real-world implementation, particularly regarding long-term diabetes management after TB treatment (2,3). These gaps were also reported in reviews assessing implementation of the WHO collaborative framework, which note that bidirectional screening is feasible but scale-up is commonly constrained by limited training, weak referral continuity, and resource constraints [41].

The implementation determinants span all five CFIR domains, highlighting the multifaceted nature of the integration challenges. While much attention has been focused on innovation characteristics and implementation processes, the inner setting (organizational context) and individual factors (provider knowledge, attitudes, and behaviours) strongly influence implementation success. This suggests that implementation strategies must address not only the procedural aspects of integration, but also the organizational and individual contexts in which integration occurs.

Workforce constraints represent the most pervasive barrier across studies, with inadequate staffing, high workload, and limited training consistently undermining integration efforts. This highlights the need for strengthening the health system as a foundation for successful TB-DM integration, rather than viewing integration solely as a clinical or programmatic initiative.

Integration approaches vary significantly in complexity and resource requirements, from simple bidirectional screening to comprehensive digital health solutions and community-based models. The concept of one size fits all doesn’t apply to all the health care settings, as the variation in implementation determinants suggests locally contextualized tailored integration strategies are needed as per the requirements of health system and facilities [42]. Despite the availability of literature focusing on the building blocks for integrated care [43], how to effectively implement integrated care still needs further exploration and expansion beyond pilot phases [44].

Patient-related factors significantly influence integration success but receive less attention in implementation frameworks than health system factors, whereas successful implementation required a holistically coordinated mechanism and pathway [45]. Swain et al. (2021) identified specific patient-related barriers including alcoholism, migrant status, old age, and lack of family support, which affected diabetes management among TB-DM comorbid patients [4]. Majumdar et al. (2019) highlighted low patient awareness about screening needs and patient dependence on doctors’ instructions as barriers to engagement [33]. Basir et al. (2019) found that financial barriers, including user fees for diagnostic tests, limited TB screening among diabetic patients in Pakistan [29]. Socioeconomic status, literacy, geographic location, and comorbidities create unique challenges for specific patient populations, necessitating tailored approaches to ensure equitable access to integrated care. The identified determinants in SAARC are similar to those found in the evidence of Asian and LMICs in general regarding the integration of TB services and NCD care [46]. A systematic review of TB-NCD integration in LMICs found that workforce capacity, diagnostic readiness, information systems, and inter-programme coordination are recurring implementation constraints, while leadership, policy alignment, and workable referral mechanisms facilitate integration closely mirroring the dominant barriers and facilitators in our synthesis [47]. A scoping review focusing on integrated TB–NCD management, with specific emphasis on diabetes, similarly highlights fragmentation of service pathways and sustainability challenges when integration is not institutionalised within routine systems [48].

The importance of digital health solutions and innovative care models is already well established to improve treatment outcomes of TB and diabetes patients [49,50,51,52], however, the same can be utilized to strengthen integrated TB comorbidities care [53]. SMARThealth enabled task-sharing between ANMs and PHC physicians in rural India [30], potentially easing overburdened services, but digital literacy gaps require context-appropriate tools, training, and support. Establishing the coordination mechanisms between TB and DM programs is another critical determinant. Considering the treatment completion of TB patients, the health system needs to establish mechanisms for the referral and continuation of diabetes services of the said patients. The fragmentation of vertical disease programs creates artificial barriers to holistic patient care, suggesting that structural health system reforms may be necessary for sustainable integration beyond project-based interventions.

Sustainability of integration efforts has emerged as a common concern across studies. Most of the studies focusing integration are either pilot or interventional studies with limited funding, which necessitates the establishment of mechanism for uptake and scale up of these interventions to ensure sustainability [8,32], [30]. Developing sustainable financing mechanisms and institutionalising integration within routine health system functions are essential for long-term impacts. In the context of SAARC countries, the health system experts, managers, policy developers and implementers need to think beyond the existing vertical programs for TB diabetes integration.

## Strength and Limitations

This review is strengthened by PROSPERO registration, PRISMA 2020 reporting, and a comprehensive search across databases plus grey literature and citation searching, designed to capture implementation evidence. We used CFIR and SWiM to structure a transparent narrative synthesis of determinants, and MMAT appraisal showed most studies were of moderate–high quality.

Limitations include evidence base unevenly distributed across the region, with studies concentrated in India and only single studies from Pakistan, Bangladesh, and Sri Lanka, and none from Afghanistan, Bhutan, Maldives, or Nepal. This limits regional representativeness and means some determinants may reflect a subset of health system contexts within SAARC. In addition, study designs and reporting were heterogeneous (quantitative descriptive, one qualitative study, and mixed methods designs), which precluded meta-analysis and required narrative synthesis; as a result, we summarised patterns of determinants rather than estimating pooled effects.

## Conclusion

This systematic review demonstrates that the successful implementation of integrating DM care into routine TB services requires a comprehensive approach that addresses determinants across all implementation domains. Although global and national frameworks guide TB–DM collaboration, sustained delivery requires context-adapted strategies that strengthen workforce capacity, coordination and referral mechanisms, standardised protocols, and monitoring systems. Digital health solutions show promise in standardising care and supporting task shifting but require adequate infrastructure and training to be effective. Patient-centred approaches that address socioeconomic and cultural factors are essential for ensuring equitable access to integrated care. Research gaps remain regarding the integration model’s cost-effectiveness, strategies for sustaining integration beyond project timeframes, and approaches to scaling up successful pilot initiatives.

### Way Forward

To further advance integrated care, we strongly recommend that policy makers and clinicians prioritize action based on the determinants outlined in this review. In particular, we will build on this evidence by conducting a qualitative study to explore implementation determinants by involving policymakers, donors, and other key stakeholders in Pakistan esp. from National and provincial TB Control Programs, National NCD program, and relevant health departments, to explore their perspectives and identify actionable solutions to the implementation of integrated diabetes care in existing TB program. Additionally, we plan to conduct both qualitative and quantitative studies at TB health facilities in at least two provinces for readiness assessment, aimed at developing evidence for the policy brief tailored to the needs of decision-makers and implementers.

## Additional Files

The additional files for this article can be found as follows:

10.5334/ijic.9882.s1 Supplementary file 1. Annexure A, B and C.

10.5334/ijic.9882.s2 Supplementary file 2. Annexure D and E.
