# Supplementary material for: Implementation Determinants of Integrated Tuberculosis and Diabetes Care in South Asian Association for Regional Cooperation (SAARC) Countries: A Systematic Review
Source: Int J Integr Care. 2026 Jun 24;26(2):21. doi: 10.5334/ijic.9882 (PMC13308530; doi:10.5334/ijic.9882)
Supplement: Supplementary file 1. — Annexure A, B and C. [file ijic-26-2-9882-s1.pdf]

Supplementary File

Annexure A

Search Strategy

1. Medline (via OVID)
2. Embase (via OVID)
3. Web of Science (WOS)
4. CINAHL
5. Cochrane Central

MEDLINE (OVID)

S.No.	Search Terms	Hits
1.	exp Tuberculosis/ or tuberculo*.mp.	(140526)
2.	Mycobacterium/ or Mycobacterium Infections/ or Mycobacterium tuberculosis/	(53092)
3.	respiratory infection.mp. or Respiratory Tract Infections/	(33497)
4.	multidrug resistant tuberculosis.mp. or Tuberculosis, Multidrug-Resistant/	(10784)
5.	TB.mp. or tb.ab,ti. or TB.af.	(70446)
6.	tuberculosis.ab,ti. or tuberculosis.af.	(147419)
7.	1 or 2 or 3 or 4 or 5 or 6	(212654)
8.	Diabetes.mp. or exp Diabetes Mellitus/ or exp diabetes mellitus/ or diabetes mellitus.af.	(614316)
9.	type 1 diabetes.mp. or Diabetes Mellitus, Type 1/	(74139)
10.	type 2 diabetes.mp. or Diabetes Mellitus, Type 2/	(206335)
11.	non-communicable disease.mp. or Noncommunicable Diseases/ or NCDs.mp.	(7939)
12.	non insulin-dependent diabetes.mp.	(4200)
13.	insulin-dependent diabetes.mp.	(9426)
14.	Blood Glucose/ or fasting blood glucose.mp.	(135908)
15.	random blood glucose.mp.	(597)
16.	oral glucose tolerance.mp. or Glucose Tolerance Test/ or OGTT.mp.	(29975)
17.	(T1D or T1DM or IDDM or IDD).mp.	(21734)
18.	(T2D or T2DM or NIDDM or NIDD).mp.	(49233)
19.	impaired glucose tolerance.mp. or Glucose Intolerance/ or *glucose/ or glucose*.af.	(455174)
20.	diabet*.mp. or diab*.af.	(743765)
21.	8 or 9 or 10 or 11 or 12 or 13 or 14 or 15 or 16 or 17 or 18 or 19 or 20	(1012551)
22.	"Delivery of Health Care, Integrated"/ or integrated care management.mp.	(14674)
23.	(integrat* or coordinat* or co-ordinat*).mp.	(812352)
24.	patient care management/ or disease management/	(41138)
25.	Integrated care pathway.mp.	(271)
26.	(management or "bidirectional screening" or screening or diagnosis or treatment or care).mp.	(8554193)
27.	co-management.mp.	(761)
28.	22 or 23 or 24 or 25 or 26 or 27	(9052682)
29.	implementation science.mp. or Implementation Science/	(5916)
30.	(implementation challenges or implementation barriers).mp.	(2680)
31.	(implement* or challeng* or determinant or barrier*).mp.	(1811949)
32.	Health Systems Challenges.mp.	(62)
33.	29 or 30 or 31 or 32	(1811949)
34.	(Asia or India or Nepal or Bangladesh or Pakistan or Bhutan or Sri Lanka or Srilanka or Maldives or Afghanistan or South Asian Association for regional cooperation or SAARC*).af.	(833166)
35.	(SAARC or SAARC countries).mp.	(78)
36.	Afghanistan.mp. or Afghanistan/	(6972)
37.	Bangladesh.mp. or Bangladesh/	(17214)
38.	Bhutan.mp. or Bhutan/	(1051)
39.	India.mp. or India/	(123958)
40.	Maldives.mp. or Maldives/	(419)
41.	Nepal.mp. or Nepal/	(12805)
42.	Pakistan.mp. or Pakistan/	(27815)
43.	Sri Lanka.mp. or Sri Lanka/	(7486)
44.	34 or 35 or 36 or 37 or 38 or 39 or 40 or 41 or 42 or 43	(833166)

45.	7 and 21 and 28 and 33 and 44	(142)

EMBASE (OVID):

S.No.	Search Terms	Hits
1.	exp Tuberculosis/ or tuberculo*.mp.	(260253)
2.	Mycobacterium/ or Mycobacterium Infections/ or Mycobacterium tuberculosis/	(91276)
3.	respiratory infection.mp. or Respiratory Tract Infections/	(68464)
4.	multidrug resistant tuberculosis.mp. or Tuberculosis, Multidrug-Resistant/	(12939)
5.	TB.mp. or tb.ab,ti. or TB.af.	(123167)
6.	tuberculosis.ab,ti. or tuberculosis.af.	(256947)
7.	1 or 2 or 3 or 4 or 5 or 6	(399800)
8.	Diabetes.mp. or exp Diabetes Mellitus/ or exp diabetes mellitus/ or diabetes mellitus.af.	(1413451)
9.	type 1 diabetes.mp. or Diabetes Mellitus, Type 1/	(147840)
10.	type 2 diabetes.mp. or Diabetes Mellitus, Type 2/	(411487)
11.	non-communicable disease.mp. or Noncommunicable Diseases/ or NCDs.mp.	(18961)
12.	non insulin-dependent diabetes.mp.	(364186)
13.	insulin-dependent diabetes.mp.	(466202)
14.	Blood Glucose/ or fasting blood glucose.mp.	(323668)
15.	random blood glucose.mp.	(1581)
16.	oral glucose tolerance.mp. or Glucose Tolerance Test/ or OGTT.mp.	(67301)
17.	(T1D or T1DM or IDDM or IDD).mp.	(46688)
18.	(T2D or T2DM or NIDDM or NIDD).mp.	(103924)
19.	impaired glucose tolerance.mp. or Glucose Intolerance/ or *glucose/ or glucose*.af.	(928701)
20.	diabet*.mp. or diab*.af.	(1603442)
21.	8 or 9 or 10 or 11 or 12 or 13 or 14 or 15 or 16 or 17 or 18 or 19 or 20	(2116201)
22.	"Delivery of Health Care, Integrated"/ or integrated care management.mp.	(14070)
23.	(integrat* or coordinat* or co-ordinat*).mp.	(1305728)
24.	patient care management/ or disease management/	(384109)
25.	Integrated care pathway.mp.	(628)
26.	(management or "bidirectional screening" or screening or diagnosis or treatment or care).mp.	(15434396)
27.	co-management.mp.	(1579)
28.	22 or 23 or 24 or 25 or 26 or 27	(16166045)
29.	implementation science.mp. or Implementation Science/	(11285)
30.	(implementation challenges or implementation barriers).mp.	(4004)
31.	(implement* or challeng* or determinant or barrier*).mp.	(3056455)
32.	Health Systems Challenges.mp.	(76)
33.	29 or 30 or 31 or 32	(3056455)
34.	(Asia or India or Nepal or Bangladesh or Pakistan or Bhutan or Sri Lanka or Srilanka or Maldives or Afghanistan or South Asian Association for regional cooperation or SAARC*).af.	(1869992)
35.	(SAARC or SAARC countries).mp.	(118)
36.	Afghanistan.mp. or Afghanistan/	(10524)
37.	Bangladesh.mp. or Bangladesh/	(26904)
38.	Bhutan.mp. or Bhutan/	(1630)
39.	India.mp. or India/	(274940)
40.	Maldives.mp. or Maldives/	(769)
41.	Nepal.mp. or Nepal/	(19486)
42.	Pakistan.mp. or Pakistan/	(50777)
43.	Sri Lanka.mp. or Sri Lanka/	(12455)
44.	34 or 35 or 36 or 37 or 38 or 39 or 40 or 41 or 42 or 43	(1869992)
45.	7 and 21 and 28 and 33 and 44	(604)

WEB OF SCIENCE

S.NO	Search Terms	Hits
1.	TS=(Tuberculosis OR tuberculo* OR mycobacterium tuberculosis OR mycobacterium infection OR respiratory tract infection OR multidrug resistant tuberculosis OR TB)	332726
2.	TS=(diabetes mellitus OR diabetes OR type 1 diabetes OR type 2 diabetes OR noncommunicable disease OR NCDs)	781475

3.	TS=(integrated care management OR integrated care pathway OR integrat* OR coordinat* OR co-ordinat* OR patient care management OR disease management OR bidirectional screening OR co-management)	3488509
4.	TS=(implementation science OR implementation challeng* OR implementation barrier* OR challenges OR barrier OR determinant OR health system challenges)	3590492
5.	TS=(Asia OR India OR Nepal OR Bangladesh OR Pakistan OR Bhutan OR Sri Lanka OR Srilanka OR Maldives OR Afghanistan OR South Asian Association for regional cooperation OR SAARC*)	619434
6.	#1 AND #2 AND #3 AND #4 AND #5	30

CINAHL

S.No	Search Terms	Results
S1	TI Tuberculosis	19,762
S2	tuberculosis or tb	37,356
S3	mycobacterium tuberculosis	6,373
S4	Tuberculosis, Multidrug-Resistant	2,664
S5	S1 OR S2 OR S3 OR S4	37,356
S6	TI Diabetes Mellitus	26,438
S7	diabetes mellitus	200,402
S8	type 1 diabetes mellitus OR diabetes mellitus type 1	31,147
S9	type 2 diabetes mellitus OR diabetes mellitus type 2	79,207
S10	noncommunicable diseases	32,215
S11	TI noncommunicable diseases	724
S12	non-insulin dependent diabetes mellitus	58,533
S13	insulin-dependent diabetes mellitus	25,388
S14	fasting blood glucose OR blood glucose OR random blood glucose	63,998
S15	oral glucose tolerance test OR glucose tolerance test OR ogtt	10,805
S16	T1DM OR IDDM OR IDD	45,844
S17	T2DM OR NIDDM OR NIDD	61,182
S18	impaired glucose tolerance OR glucose intolerance	9,360
S19	diabet*	296,275
S20	S6 OR S7 OR S8 OR S9 OR S10 OR S11 OR S12 OR S13 OR S14 OR S15 OR S16 OR S17 OR S18 OR S19	370,977
S21	integrated care management OR integrated care pathway	1,431
S22	patient care management	7,844
S23	disease management	71,215
S24	integrat* OR "integrated"	190,025
S25	"bidirectional screening" OR screening	226,161
S26	coordinat* OR co-ordinat*	54,131
S27	co-management	386
S28	S21 OR S22 OR S23 OR S24 OR S25 OR S26 OR S27	522,024
S29	implementation science OR implementation	175,902
S30	implementation challeng* OR implementation barrier*	11,894
S31	challeng* OR implement* OR barrier* OR determinant	726,725
S32	health system challenges	2,607
S33	S29 OR S30 OR S31 OR S32	726,725
S34	TI (Asia or India or Nepal or Bangladesh or Pakistan or Bhutan or Sri Lanka or Srilanka or Maldives or Afghanistan or South Asian Association for regional cooperation or SAARC*)	43,927
S35	SAARC OR SAARC countries	23
S36	Afghanistan	4,660
S37	Bangladesh	7,084
S38	Bhutan	478
S39	India	62,483
S40	Maldives	117
S41	Nepal	4,908
S42	Pakistan	8,757
S43	sri lanka	3,438
S44	S34 OR S35 OR S36 OR S37 OR S38 OR S39 OR S40 OR S41 OR S42 OR S43	92,800
S45	S5 AND S20 AND S28 AND S33 AND S44	7

Cochrane CENTRAL

S.No	Search Terms	Results
#1	MeSH descriptor: [Tuberculosis] explode all trees	3472

#2	("tuberculosis"):ti,ab,kw	8336
#3	MeSH descriptor: [Mycobacterium tuberculosis] explode all trees	504
#4	Tuberculosis, Multidrug-Resistant	581
#5	(TB):ti,ab,kw	4644
#6	#1 OR #2 OR #3 OR #4 OR #5	9980
#7	MeSH descriptor: [Diabetes Mellitus] explode all trees	46298
#8	("diabetes mellitus"):ti,ab,kw	86515
#9	"type 1 diabetes mellitus" OR Diabetes Mellitus Type 1	67769
#10	"type 2 diabetes mellitus" OR Diabetes Mellitus Type 2	74932
#11	MeSH descriptor: [Noncommunicable Diseases] explode all trees	125
#12	(noncommunicable disease):ti,ab,kw	763
#13	("non-insulin-dependent diabetes"):ti,ab,kw	22534
#14	("insulin dependent diabetes"):ti,ab,kw	27589
#15	fasting blood glucose OR "blood glucose" OR random blood glucose	52845
#16	oral glucose tolerance OR glucose tolerance test OR OGTT	10477
#17	(T1D OR T1DM OR IDDM OR IDD):ti,ab,kw	4483
#18	(T2D OR T2DM OR NIDDM OR NIDD):ti,ab,kw	15924
#19	impaired glucose tolerance OR "glucose intolerance"	6119
#20	diabet*	132862
#21	#7 OR #8 OR #9 OR #10 OR #11 OR #12 OR #13 OR #14 OR #15 OR #16 OR #17 OR #18 OR #19 OR #20	155515
#22	(integrated care management):ti,ab,kw	3180
#23	(integrated care pathway):ti,ab,kw	280
#24	(patient care management):ti,ab,kw	36257
#25	("disease management"):ti,ab,kw	16946
#26	(integrat* OR "integrated"):ti,ab,kw	39760
#27	("bidirectional screening" OR screening):ti,ab,kw	83513
#28	(coordinat* OR co-ordinat*):ti,ab,kw	15897
#29	(co-management):ti,ab,kw	75
#30	#22 OR #23 OR #24 OR #25 OR #26 OR #27 OR #28 OR #29	173799
#31	(implementation science OR implementation):ti,ab,kw	36026
#32	(implementation challeng* OR implementation barrier*):ti,ab,kw	8287
#33	(challeng* OR implement* OR barrier* OR determinant):ti,ab,kw	137296
#34	(health system challenges):ti,ab,kw	1620
#35	#31 OR #32 OR #33 OR #34	137296
#36	(Asia or India or Nepal or Bangladesh or Pakistan or Bhutan or Sri Lanka or Srilanka or Maldives or Afghanistan or South Asian Association for regional cooperation or SAARC*):ti,ab,kw	25743
#37	(SAARC or SAARC countries)	9
#38	#36 OR #37	25753
#39	#6 AND #21 AND #30 AND #35 AND #38	6

Annexure B

Mixed Methods Appraisal Tool (MMAT), version 2018

**Category of study designs**	**Methodological quality criteria**	**Responses**			
		Yes	No	Can't tell	Comments
Screening questions (for all types)	S1. Are there clear research questions?				
	S2. Do the collected data allow to address the research questions?				
	Further appraisal may not be feasible or appropriate when the answer is 'No' or 'Can't tell' to one or both screening questions.				
1. Qualitative	1.1. Is the qualitative approach appropriate to answer the research question?				
	1.2. Are the qualitative data collection methods adequate to address the research question?				
	1.3. Are the findings adequately derived from the data?				
	1.4. Is the interpretation of results sufficiently substantiated by data?				
	1.5. Is there coherence between qualitative data sources, collection, analysis and interpretation?				
2. Quantitative randomized controlled trials	2.1. Is randomization appropriately performed?				
	2.2. Are the groups comparable at baseline?				
	2.3. Are there complete outcome data?				
	2.4. Are outcome assessors blinded to the intervention provided?				
	2.5 Did the participants adhere to the assigned intervention?				
3. Quantitative non-randomized	3.1. Are the participants representative of the target population?				
	3.2. Are measurements appropriate regarding both the outcome and intervention (or exposure)?				
	3.3. Are there complete outcome data?				
	3.4. Are the confounders accounted for in the design and analysis?				
	3.5. During the study period, is the intervention administered (or exposure occurred) as intended?				
4. Quantitative descriptive	4.1. Is the sampling strategy relevant to address the research question?				
	4.2. Is the sample representative of the target population?				
	4.3. Are the measurements appropriate?				
	4.4. Is the risk of nonresponse bias low?				
	4.5. Is the statistical analysis appropriate to answer the research question?				
5. Mixed methods	5.1. Is there an adequate rationale for using a mixed methods design to address the research question?				
	5.2. Are the different components of the study effectively integrated to answer the research question?				
	5.3. Are the outputs of the integration of qualitative and quantitative components adequately interpreted?				
	5.4. Are divergences and inconsistencies between quantitative and qualitative results adequately addressed?				
	5.5. Do the different components of the study adhere to the quality criteria of each tradition of the methods involved?				

Annexure C

MMAT Evaluation – Quantitative Descriptive Study

Basir et al. (2019)

MMAT Criterion	Yes (✓)	No (✗)	Can't tell	Explanation	Score
4.1 Is the sampling strategy relevant to address the research question?	✓			The study screened all patients attending clinics, appropriate for assessing feasibility.	1
4.2 Is the sample representative of the target population?	✓			Large and geographically diverse sample from Karachi, likely representative.	1
4.3 Are the measurements appropriate?	✓			Used standard tools (RBS, Xpert MTB/RIF, CXR) appropriate for diagnosis.	1
4.4 Is the risk of nonresponse bias low?		✓		Did not report refusals or missed screenings—unclear nonresponse management.	0
4.5 Is the statistical analysis appropriate to answer the research question?	✓			Basic descriptive statistics used and clearly reported.	1

Total Score: 4/5

Satyanarayana et al. (2013)

MMAT Criterion	Yes (✓)	No (✗)	Can't tell	Explanation	Score
4.1 Is the sampling strategy relevant to address the research question?	✓			TB patients were screened for diabetes in selected tertiary and peripheral institutions across India—appropriate for feasibility assessment.	1
4.2 Is the sample representative of the target population?	✓			Inclusion of multiple levels of healthcare (tertiary and peripheral) increases representativeness.	1
4.3 Are the measurements appropriate?	✓			Blood glucose testing methods were consistent with clinical standards.	1
4.4 Is the risk of nonresponse bias low?		✓		The study does not provide enough detail on how many eligible patients declined or missed screening.	0
4.5 Is the statistical analysis appropriate to answer the research question?	✓			Descriptive statistics used appropriately to report screening results.	1

Total Score: 4/5

Rafi et al. (2024)

MMAT Criterion	Yes (✓)	No (✗)	Can't tell	Explanation	Score
4.1 Is the sampling strategy relevant to address the research question?	✓			Stratified random sampling from formal-sector health facilities was relevant for assessing readiness.	1
4.2 Is the sample representative of the target population?	✓			Large, nationally representative sample of TB care facilities used.	1
4.3 Are the measurements appropriate?	✓			Readiness and availability assessed using WHO-SARA tools—highly appropriate.	1
4.4 Is the risk of nonresponse bias low?	✓			Response rate was 97%, indicating minimal risk of bias.	1

Total Score: 5/5

Rajapakshe et al. (2015)

MMAT Criterion	Yes (✓)	No (✗)	Can't tell	Explanation	Score
4.1 Is the sampling strategy relevant to address the research question?	✓			Total sampling of registered TB patients in a defined clinic during the study period was used.	1
4.2 Is the sample representative of the target population?	✓			All eligible TB patients in the Ampara district were considered, enhancing representativeness.	1
4.3 Are the measurements appropriate?	✓			FBG testing was conducted as per clinical diagnostic guidelines.	1
4.4 Is the risk of nonresponse bias low?		✓		Nonresponse bias exists as 21 out of 104 eligible patients did not undergo FBG testing.	0

Total Score: 3/4

Kunjathur et al., (2025)

MMAT Criterion	Yes (✓)	No (✗)	Can't tell	Explanation	Score
4.1 Is the sampling strategy relevant to address the research question?	✓			All presumptive TB patients in selected centers were sampled.	1
4.2 Is the sample representative of the target population?	✓			Large dataset from Bengaluru covers a broad target population.	1
4.3 Are the measurements appropriate?	✓			Standard blood glucose tests were used per clinical norms.	1
4.4 Is the risk of nonresponse bias low?		✓		FBS follow-up was limited; some patients may not have been tested.	0
4.5 Is the statistical analysis appropriate to answer the research question?	✓			Descriptive statistics were appropriately used for findings.	1

Total Score: 4/5

MMAT Evaluation – Qualitative Study

Koya et al. (2022)

MMAT Criterion	Yes (✓)	No (✗)	Can't tell	Explanation	Score
1.1 Are the research questions clear and aligned with the qualitative approach?	✓			Research questions focused on system-level barriers and stakeholder perspectives—suitable for qualitative inquiry.	1
1.2 Is the qualitative approach appropriate to answer the research question?	✓			Use of FGDs and IDIs with diverse stakeholders was appropriate for exploring implementation challenges.	1
1.3 Are the data collection methods adequate to address the research question?	✓			Detailed qualitative data were collected through structured interviews and discussions.	1
1.4 Are the findings substantiated by the data?	✓			Themes were well-supported with quotations and examples from stakeholders.	1
1.5 Is the interpretation of results sufficiently supported by the data?	✓			Interpretations were logically drawn and aligned with the qualitative data presented.	1

Total Score: 5/5

MMAT Evaluation – All Mixed Methods Studies (5+1+4)

Anand et al. (2018)

Section 1 – Qualitative

MMAT Criterion	Yes	No	Can't tell
1.1 Is the qualitative approach appropriate?	✓		
1.2 Are the qualitative data collection methods adequate?	✓		
1.3 Are the findings derived from the data?	✓		
1.4 Is the interpretation of results substantiated by the data?	✓		
1.5 Is there coherence between qualitative data sources, collection, analysis and interpretation?	✓		

Section 4 – Quantitative Descriptive

MMAT Criterion	Yes	No	Can't tell
4.1 Is the sampling strategy relevant to address the research question?	✓		
4.2 Is the sample representative of the target population?	✓		
4.3 Are the measurements appropriate?	✓		
4.4 Is the risk of nonresponse bias low?	✓		
4.5 Is the statistical analysis appropriate to answer the research question?	✓		

Section 5 – Mixed Methods

MMAT Criterion	Yes	No	Can't tell
5.1 Is there an adequate rationale for using a mixed methods design?	✓		
5.2 Are the different components of the study effectively integrated?	✓		

5.3 Are the outputs of the integration of qualitative and quantitative components adequately interpreted?	✓		
5.4 Are divergences and inconsistencies between quantitative and qualitative results adequately addressed?	✓		
5.5 Do the different components of the study adhere to the quality criteria of each tradition of the methods involved?	✓		

Swain et al. (2021)

Section 1 – Qualitative

MMAT Criterion	Yes	No	Can't tell
1.1 Is the qualitative approach appropriate?	✓		
1.2 Are the qualitative data collection methods adequate?	✓		
1.3 Are the findings derived from the data?	✓		
1.4 Is the interpretation of results substantiated by the data?	✓		
1.5 Is there coherence between qualitative data sources, collection, analysis and interpretation?			✓

Section 4 – Quantitative Descriptive

MMAT Criterion	Yes	No	Can't tell
4.1 Is the sampling strategy relevant to address the research question?	✓		
4.2 Is the sample representative of the target population?	✓		
4.3 Are the measurements appropriate?	✓		
4.4 Is the risk of nonresponse bias low?	✓		

4.5 Is the statistical analysis appropriate to answer the research question?	✓		

Section 5 – Mixed Methods

MMAT Criterion	Yes	No	Can't tell
5.1 Is there an adequate rationale for using a mixed methods design?	✓		
5.2 Are the different components of the study effectively integrated?	✓		
5.3 Are the outputs of the integration of qualitative and quantitative components adequately interpreted?	✓		
5.4 Are divergences and inconsistencies between quantitative and qualitative results adequately addressed?	✓		
5.5 Do the different components of the study adhere to the quality criteria of each tradition of the methods involved?	✓		

Joshi et al. (2022)

Section 1 – Qualitative

MMAT Criterion	Yes	No	Can't tell
1.1 Is the qualitative approach appropriate?	✓		
1.2 Are the qualitative data collection methods adequate?	✓		
1.3 Are the findings derived from the data?	✓		
1.4 Is the interpretation of results substantiated by the data?	✓		
1.5 Is there coherence between qualitative data sources, collection, analysis and interpretation?			✓

Section 2 – RCT

MMAT Criterion	Yes	No	Can't tell
2.1 Was randomization appropriately performed?	✓		
2.2 Were the groups comparable at baseline?	✓		
2.3 Were there complete outcome data?	✓		
2.4 Were outcome assessors blinded to the intervention?	✓		
2.5 Did the participants adhere to the assigned intervention?	✓		

Section 5 – Mixed Methods

MMAT Criterion	Yes	No	Can't tell
5.1 Is there an adequate rationale for using a mixed methods design?	✓		
5.2 Are the different components of the study effectively integrated?	✓		
5.3 Are the outputs of the integration of qualitative and quantitative components adequately interpreted?	✓		
5.4 Are divergences and inconsistencies between quantitative and qualitative results adequately addressed?	✓		
5.5 Do the different components of the study adhere to the quality criteria of each tradition of the methods involved?	✓		

Majumdar et al. (2019)

Section 1 – Qualitative

MMAT Criterion	Yes	No	Can't tell
1.1 Is the qualitative approach appropriate?	✓		
1.2 Are the qualitative data	✓		

collection methods adequate?			
1.3 Are the findings derived from the data?	✓		
1.4 Is the interpretation of results substantiated by the data?		✓	
1.5 Is there coherence between qualitative data sources, collection, analysis and interpretation?	✓		

Section 4 – Quantitative Descriptive

MMAT Criterion	Yes	No	Can't tell
4.1 Is the sampling strategy relevant to address the research question?	✓		
4.2 Is the sample representative of the target population?	✓		
4.3 Are the measurements appropriate?	✓		
4.4 Is the risk of nonresponse bias low?		✓	
4.5 Is the statistical analysis appropriate to answer the research question?	✓		

Section 5 – Mixed Methods

MMAT Criterion	Yes	No	Can't tell
5.1 Is there an adequate rationale for using a mixed methods design?	✓		
5.2 Are the different components of the study effectively integrated?	✓		
5.3 Are the outputs of the integration of qualitative and quantitative components adequately interpreted?	✓		
5.4 Are divergences and inconsistencies between quantitative and qualitative results adequately addressed?	✓		
5.5 Do the different components of the	✓		

study adhere to the quality criteria of each tradition of the methods involved?			
